# The Diagnostic Accuracy of Colon Capsule Endoscopy in Inflammatory Bowel Disease—A Systematic Review and Meta-Analysis

**DOI:** 10.3390/diagnostics14182056

**Published:** 2024-09-16

**Authors:** Ian Io Lei, Camilla Thorndal, Muhammad Shoaib Manzoor, Nicholas Parsons, Charlie Noble, Cristiana Huhulea, Anastasios Koulaouzidis, Ramesh P. Arasaradnam

**Affiliations:** 1 Institute of Precision Diagnostics & Translational Medicine, University Hospital of Coventry and Warwickshire, Clifford Bridge Rd, Coventry CV2 2DX, UK; cristiana.huhulea@uhcw.nhs.uk (C.H.); r.arasaradnam@warwick.ac.uk (R.P.A.); 2 Warwick Medical School, University of Warwick, Coventry CV4 7AL, UK; 3 Surgical Research Unit, Odense University Hospital, 5700 Svendborg, Denmark; camilla.thorndal.nielsen@rsyd.dk (C.T.); akoulaouzidis@hotmail.com (A.K.); 4 Department of Gastroenterology, Sandwell and West Birmingham Hospitals NHS Trust, Hallam St., West Bromwich B71 4HJ, UK; muhammad_shoaib_manzoor@yahoo.com; 5 Warwick Clinical Trials Unit, University of Warwick, Coventry CV4 7AL, UK; nick.parsons@warwick.ac.uk; 6 Noblesoft, Stamford, Lincs PE9 3DN, UK; c.noble@noblesoft.co.uk; 7 Department of Clinical Research, University of Southern Denmark, 5230 Odense, Denmark; 8 Department of Gastroenterology, Pomeranian Medical University, 70-204 Szczecin, Poland; 9 Department of Surgery, OUH Svendborg Sygehus, 5700 Svendborg, Denmark; 10 Leicester Cancer Centre, University of Leicester, Leicester LE1 7RH, UK

**Keywords:** colon capsule endoscopy, panenteric capsule endoscopy, inflammatory bowel disease, non-polypoidal colonic conditions, colonic inflammation, colonic inflammatory conditions

## Abstract

Colon capsule endoscopy (CCE) has regained popularity for lower gastrointestinal investigations since the COVID-19 pandemic. While there have been systematic reviews and meta-analyses on colonic polyp detection using CCE, there is a lack of comprehensive evidence concerning colonic inflammation. Therefore, this systematic review and meta-analysis aimed to assess the diagnostic accuracy of CCE for colonic inflammation, predominantly ulcerative colitis (UC) and Crohn’s disease (CD). **Methods:** We systematically searched electronic databases (EMBASE, MEDLINE, PubMed Central, and Cochrane Library) for studies comparing the diagnostic accuracy between CCE and optical endoscopy as the standard reference. A bivariate random effect model was used for the meta-analysis. **Results:** From 3797 publications, 23 studies involving 1353 patients were included. Nine studies focused on UC, and ten focused on CD. For UC, CCE showed a pooled sensitivity of 92% (95% CI, 88–95%), a specificity of 71% (95% CI, 35–92%), and an AUC of 0.93 (95% CI, 0.89–0.97). For CD, the pooled sensitivity was 92% (95% CI, 89–95%), and the specificity was 88% (95% CI, 84–92%), with an AUC of 0.87 (95% CI, 0.76–0.98). Overall, for inflammatory bowel disease, the pooled sensitivity, specificity, and AUC were 90% (95% CI, 85–93%), 76% (95% CI, 56–90%), and 0.92 (95% CI, 0.94–0.97), respectively. **Conclusions:** Despite the challenges around standardised disease scoring and the lack of histological confirmation, CCE performs well in diagnosing inflammatory bowel disease. It demonstrates high sensitivity in both UC and Crohn’s terminal ileitis and colitis and high specificity in Crohn’s disease. Further studies are needed to evaluate the diagnostic accuracy of other colonic inflammatory conditions.

## 1. Introduction

In recent years, colon capsule endoscopy (CCE) has gained significant popularity as an alternative to colonoscopy and computed tomography colonography (CTC) for lower gastrointestinal (GI) investigations. CCE witnessed widespread adoption in the Scottish, English, and Danish healthcare systems, producing large-scale studies such as ScotCap, NHS England Pilot, and CareForColon [1,2]. Several systematic reviews were published but focused only on polyps and colorectal cancer (CRC) detection using CCE [3,4,5]. Moreover, a systematic review of artificial intelligence (AI) in CCE indicated that all the attention was given to polyp detection [6].

In comparison, other colonic inflammatory conditions such as inflammatory bowel disease (IBD), including ulcerative colitis (UC) and Crohn’s disease (CD), have not been studied to the same extent in CCE. Even though CD has been studied extensively in small-bowel capsule endoscopy (SBCE) in the literature, CCE is radically different from SBCE, especially since CCE has two cameras rather than the one in the SB capsule [7]. Therefore, the diagnostic accuracy of CCE in UC and CD remains unclear, resulting in the absence of relevant recommendations for using CCE for suspected IBD [8]. In the literature, the latest systematic review was conducted by Tamilarasan et al. on the diagnostic accuracy of IBD using “panenteric capsule endoscopy (PCE)—Crohn’s capsule^TM^ (Medtronic, Minneapolis, MN, USA)” rather than the newest model of colon capsule endoscopy—CCE 2 (PillCam Colon 2^TM^, Medtronic, Minneapolis, MN, USA). The Crohn’s capsule is derived from the reprogrammed and software-redesigned CCE2, addressing a limitation of the original CCE1—the inability to capture the entire small bowel fully. Given the similarity between CCE2 and Crohn’s capsule, that systematic review also employed studies including CCE 1 and 2. However, significant emphasis was placed on small-bowel CD, magnetic resonance enterography (MRE), and PCE instead of CCE [9].

As the utilisation of CCE becomes more widespread, various colonic inflammatory conditions have become more apparent. Therefore, it is crucial to understand the diagnostic accuracy of CCE for these conditions. In addition, a non-invasive diagnostic alternative to colonoscopy would also be beneficial, as colonoscopy can cause significant discomfort. This is especially relevant for IBD patients, who often require frequent surveillance colonoscopies. Therefore, this systematic review and meta-analysis will assess the diagnostic accuracy of inflammatory colonic conditions, predominantly UC and terminal ileal (TI) and colonic CD activity, using CCE.

## 2. Methods

The study protocol was designed based on PRISMA-DTA recommendations [10,11]. The primary aim of the review was to evaluate the per-patient diagnostic accuracy in identifying active UC and terminal ileal and colonic CD using CCE compared to ileocolonoscopy (IC). The secondary aims include assessing the pooled correlation of the detection of IBD severity between CCE and colonoscopy and the diagnostic yield of other miscellaneous pathologies such as diverticular disease, telangiectasia, and haemorrhoids.

### 2.1. Eligibility Criteria

The search included all full texts of clinical and prospective trials that evaluated the diagnostic accuracy of CCE in patients with colonic inflammatory pathologies without language restrictions. This included both adult and paediatric studies. A clear comparison of CCE with IC or transanal enteroscopy as a reference standard is required. There was no restriction on the CCE patients’ recruiting criteria in these studies. Conference abstracts were not included due to the high risk of bias [12]. Review articles, systematic reviews, editorials, study protocols, case reports, and small case series or studies involving ≤10 participants were also excluded.

### 2.2. Information Sources

The databases used to identify relevant publications included EMBASE, MEDLINE, PubMed Central, and Cochrane Library. Additional publications were hand-searched using the references of the extracted studies. The electronic search included all studies up to 8 September 2023, but the search was conducted without any additional time limitations. The search comprised MeSH and non-MeSH terms, including IBD, UC, CD, diverticulitis, infective colitis, checkpoint inhibitor colitis, lower GI bleed, telangiectasia, radiation, and microscopic colitis (see Appendix A, Table A1). The search strings used for each database are available in Appendix A. Grey literature, and unpublished studies were not included.

### 2.3. Study Selection

The title and abstract of all the retrieved studies were reviewed by three of the authors (I.L., C.T., and M.S.M.), and all the studies that did not meet the eligibility criteria were excluded. The inclusion criteria used in the subsequent full-text review were as follows:

- Comparison between CCE (including both CCE1 or CCE2 or Crohn’s capsule only for terminal ileal and colonic findings) and IC as the comparator arm.
- The interval between CCE and subsequent IC must be within two weeks.
- Any colonic inflammation in non-polypoidal pathologies.
- A prospective study with >10 participants.
- Use detection or diagnosis of these pathologies as the predominant study endpoint.

**Figure 1:**
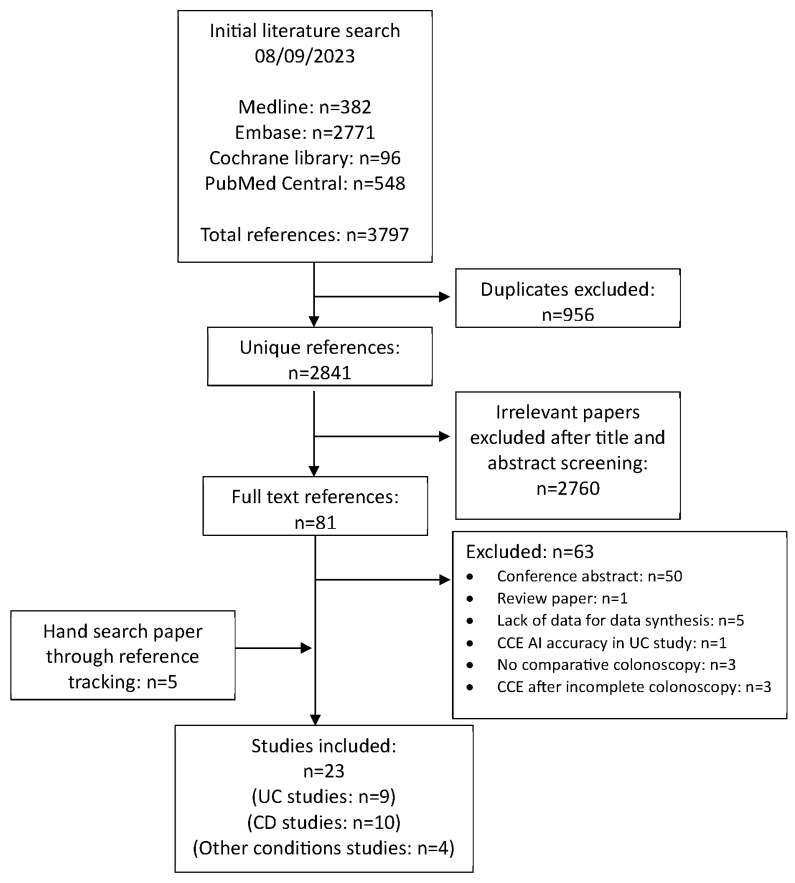
PRISMA flow chart [1].

The exclusion criteria are predominantly polyps, CRC, and the use of a small-bowel capsule (see Figure 1). Therefore, five diagnostic studies with inadequate data for data synthesis were excluded from the final analysis but are listed for reference within the appendix (see Appendix A, Table A6).

### 2.4. Data Compilation

The final selection of studies was then reviewed, and data were extracted. The details included the type of pathologies, accuracy, assessment score, severity assessment, extent of disease assessment, type of capsule, comparator arm, regimen of bowel preparation such as the use of prokinetic drugs and boosters, study type, sample size, bowel cleansing quality, and CCE procedure completion rate (see Appendix A, Table A2, Table A3 and Table A4).

### 2.5. Risk of Bias

**Figure A1:**
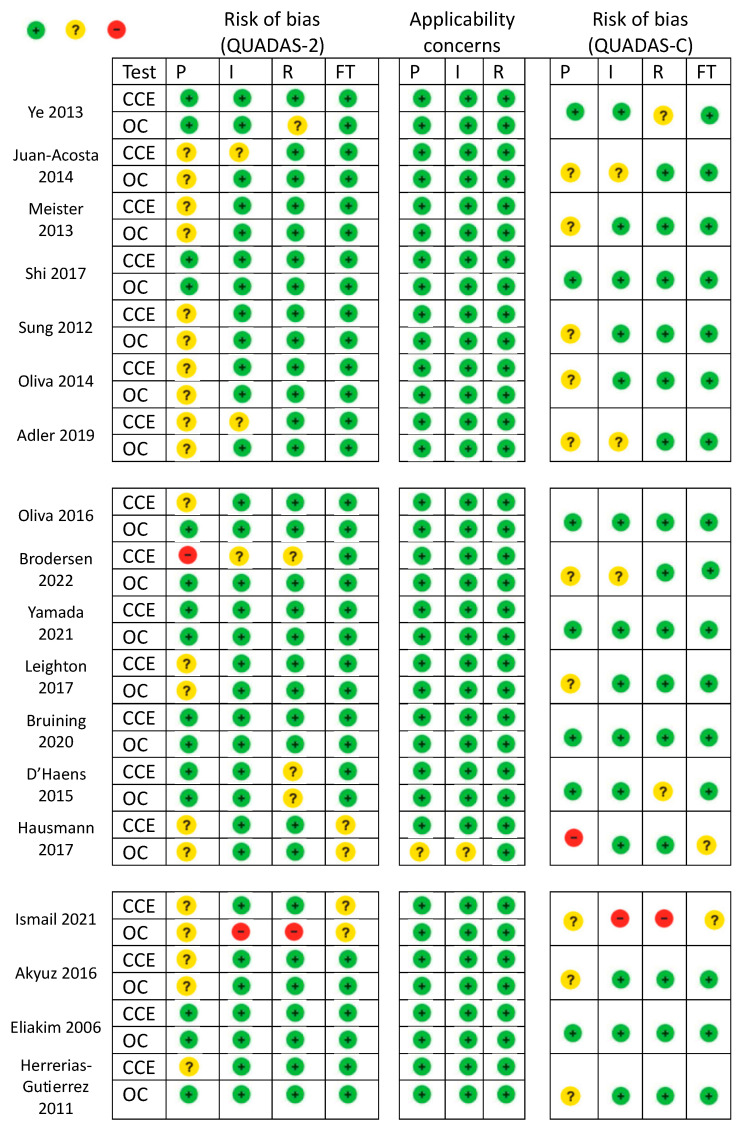
Risk-of-bias assessment using the QUADAS 2 and QUADAS-C tools. Leighton 2017 [46], Ye 2013 [28], Juan-Acosta 2014 [29], Shi 2017 [30], Adler 2019 [31], Oliva 2014 [32] Sung 2012 [33], Meister 2013 [34], Oliva 2016 [37], Brodersen 2022 [38], Hausmann 2017 [39], Bruining 2020 [40], Yamada 2021 [41], D’Haens 2015 [44], Ismail 2021 [53], Akyuz 2016 [54], Eliakim 2006 [55], Herrerias-Gutierrez 2011 [56].

The selected studies underwent a risk-of-bias assessment utilising the Quality Assessment of Diagnostic Accuracy in Systematic Review-2 and Comparative Study (QUADAS-2 and QUADAS-C) as a component of the quality assurance procedure [13,14]. The risk of bias and the applicability were categorised as low, unclear, or high (see Appendix A, Figure A1).

### 2.6. Data Synthesis and Statistical Analysis

The confusion matrices, which provide the number of true positives (TPs), true negatives (TNs), false positives (FPs), and false negatives (FNs), were extracted from the data provided in each study and categorised into ulcerative colitis (UC), Crohn’s disease (CD), and other pathologies. The key quantitative metrics derived from each study were the sensitivity, specificity, positive predictive value (PPV), and negative predictive value (NPV) [15] (see Figure 1). A diagnostic test accuracy meta-analysis was conducted using a bivariate logistic regression model with random effects (bivariate GLMM) [16,17]. This model, based on pairs of TPs, TNs, FPs, and FNs, was used to calculate pooled estimates of the sensitivity, specificity, diagnostic odds ratio (DOR), positive likelihood ratio (PLR), negative likelihood ratio (NLR), and area under the curve (AUC) using a summary receiver operating characteristic (sROC) curve. The PLR indicates the likelihood that a patient has IBD given a positive CCE result. At the same time, the NLR reflects the likelihood that a patient with a negative CCE result actually has the condition. The AUC serves as a global measure of test performance, with diagnostic accuracy classified as low (AUC < 0.7), moderate (0.7 ≤ AUC < 0.9), or high (AUC ≥ 0.9) [18]. The heterogeneity variance of the logit-transformed sensitivity and specificity of the model and forest plots were used to visualise and explore potential sources of heterogeneity between studies for both sensitivity and specificity. The meta-analysis was carried out using the “lme4” (version 1.1-35.5) [17], “msm” (version 1.8) [19], “mada” (version 0.5.11) [20], and ”lmtest”(version 1.0.0) [21] R packages within the R software (R Core Team, R Foundation for Statistical Computing, Vienna, Austria, https://www.R-project.org/, 25 August 2024) [22]. The correlation coefficients between CCE and ileocolonoscopy in IBD disease detection and disease activity were also reported as a secondary outcome.

### 2.7. Subgroup Analysis and Sensitivity Analysis

When four or more studies were available for subgroup analyses, a meta-analysis was performed following the methods recommended by the Cochrane Diagnostic Test Accuracy Working Group [23]. For studies focusing on CD, the subgroup analysis was limited to two segments: TI and overall colonic disease. In the subgroup and sensitivity analysis, we incorporated pre-defined subgroups, including the CCE1 and CCE2 capsules, Crohn’s capsule and CCE2, different disease extent (CD terminal ileitis vs. CD colitis), different prokinetics usage (such as domperidone, metoclopramide, or none), and various bowel preparation methods (combination of polyethylene glycol, magnesium citrate, senna, and bisacodyl), completion rate (<70%, 70–90%, and >90%), and cleansing quality (adequacy < 70%, 70–90%, and >90%). Post hoc subgroup analysis detected heterogeneity and diagnostic yield variations in these pre-defined categories. 

### 2.8. Publication Bias

In order to assess if systematic differences among the relevant selected studies were missed, we adopted an effective sample-size funnel plot together with the associated regression test of asymmetry, which was the Deeks funnel-plot asymmetry test using DOR and effective sample size (ESS) [24].

## 3. Results

### 3.1. Literature Search

A total of 3797 references from four different databases were identified from the literature search (see Figure 1) [25]. CD studies that assessed the diagnostic accuracy of small-bowel CD only or without IC as a comparator were excluded. Five diagnostic studies did not have adequate data for data synthesis, so they were excluded from the analysis. Twenty-three studies were included in the final analysis, and all the relevant data were extracted by I.L. (see Appendix A, Table A2, Table A3, Table A4 and Table A5) [26,27,28,29,30,31,32,33,34,35,36,37,38,39,40,41,42,43].

### 3.2. Study Characteristics

The selected studies were published between 2006 and 2023, reporting a total number of 1353 enrolled patients. The studies were categorised into UC studies (*n* = 458), CD studies (*n* = 665), and other pathology studies (*n* = 230); overall summaries of the study methodology and results are presented in Table A2 and Table A3 (Appendix A). Due to the limited number of studies on other colonic inflammatory conditions, this systematic review predominantly focuses on inflammatory bowel disease. The findings of other non-polypoidal conditions beyond the scope of IBD are summarised in Table A5 (Appendix A).

Seven of the nine UC studies had adequate data for calculating the diagnostic accuracy. In addition, seven studies reported results utilising the correlation coefficient in UC disease activity and severity detection. In CD studies, six out of nine studies had adequate data for calculating the diagnostic accuracy, and seven studies assessed the overall correlation coefficient in Crohn’s disease detection in the colon and terminal ileum. One of the CD studies used transanal double-balloon enteroscopy as a comparator, and the rest used IC as the gold standard. 

For capsule type, the distribution across studies was as follows: CCE1 (*n* = 5), CCE2 (*n* = 12), both CCE1 and CCE2 (*n* = 1), unspecified CCE type (*n* = 1), and PillCam Crohn’s capsule (*n* = 5) (see Appendix A, Table A4). During the data synthesis, three studies [31,34,44] performed sensitivity, specificity, PPV, and NPV calculations from the raw data. 

For the disease activity assessment score in UC studies, the Mayo score (*n* = 4), UCEIS (*n* = 2), Matt’s endoscopic score (*n* = 2), and Rachmilewitz score (*n* = 1) were used. For the CD studies, the Simple Endoscopic Score (SES-CD) (*n* = 7), Lewis score (*n* = 3), CACDAI score (*n* = 1), and Rugeerts score (*n* = 1) were used. 

### 3.3. Bowel Cleansing and Capsule Completion Rates

Bowel cleansing is pivotal for CCE as it affects diagnostic accuracy. Detailed bowel preparation protocols were reported across all studies, where polyethene glycol (PEG)-based preparation was universally used. Five studies also incorporated additional laxatives as part of their bowel preparation regimen, with notable variations among them. However, four studies did not include the doses. Evaluating whether bowel preparation scores were consistently reported was performed using a 4-point scoring system, including poor, fair, good, and excellent. Only one study did not provide any cleansing scores. The adequacy of overall bowel preparation, defined as fair or above, revealed varying percentages across different studies, ranging from 49% to 98.5% [45].

Another critical performance determinant was the completion rate, which ranged from 68% to 100%. One study did not report the completion rate (see Appendix A, Table A4). 

### 3.4. Risk-of-Bias and Publication Bias Assessment

**Figure A2:**
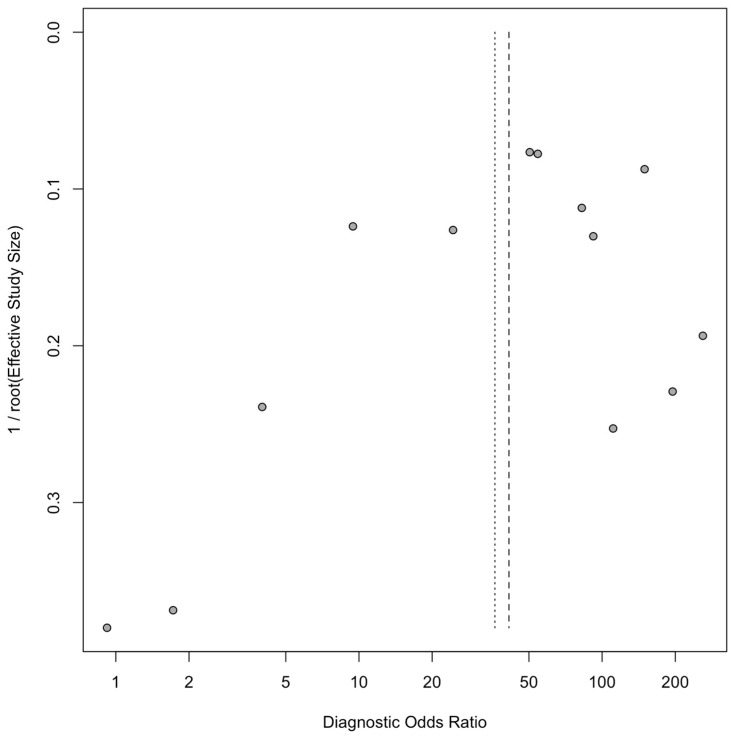
Funnel plot for diagnostic odds ratios—Deeks’ regression test for publication bias. Test result: t = −1.26; df = 11; *p*-value = 0.2329. Bias estimate: −6.4309 (standard error = 5.0937); multiplicative residual heterogeneity variance (tau^2^ = 66.0431).

The studies’ risk-of-bias assessments based on the QUADAS 2 and QUADAS-C tools are presented in Appendix A (Figure A1). Three studies were classified as at high risk of bias, while eleven were identified with an unclear risk of bias. The risk of bias in these studies mainly originated from inadequate specification of the patient selection criteria or unblinding during the selection process, the degree of blinding in the endoscopists and CCE readers, the prolonged period between the index and reference tests, and unclear allocation sequences. Deeks’ regression test showed a *p*-value = 0.23, which suggested no evidence of publication bias (see Appendix A, Figure A2).

### 3.5. Diagnostic Accuracy

**Figure 2:**
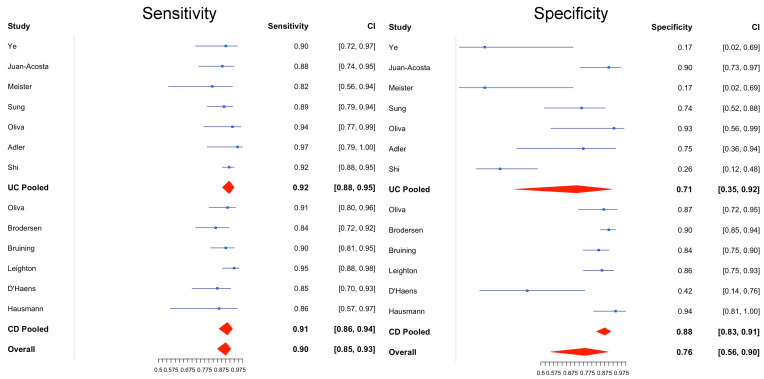
The forest plot of sensitivity (left) and specificity (right) of IBD (including both UC and CD disease activity) detection using CCE. Leighton 2017 [46], Ye C. A. 2013 [28], Juan-Acosta 2014 [29], Shi 2017 [30], Adler 2019 [31], Oliva 2014 [32] Sung 2012 [33], Meister 2013 [34], Hosoe 2013 [35], Hosoe 2018 [36], Oliva 2016 [37], Brodersen 2022 [38], Hausmann 2017 [39], Bruining 2020 [40], Yamada 2021 [41], Papalia 2021 [42], Brodersen 2023 [43], Hall 2015 [47], D’Haens 2015 [44].

**Figure 3:**
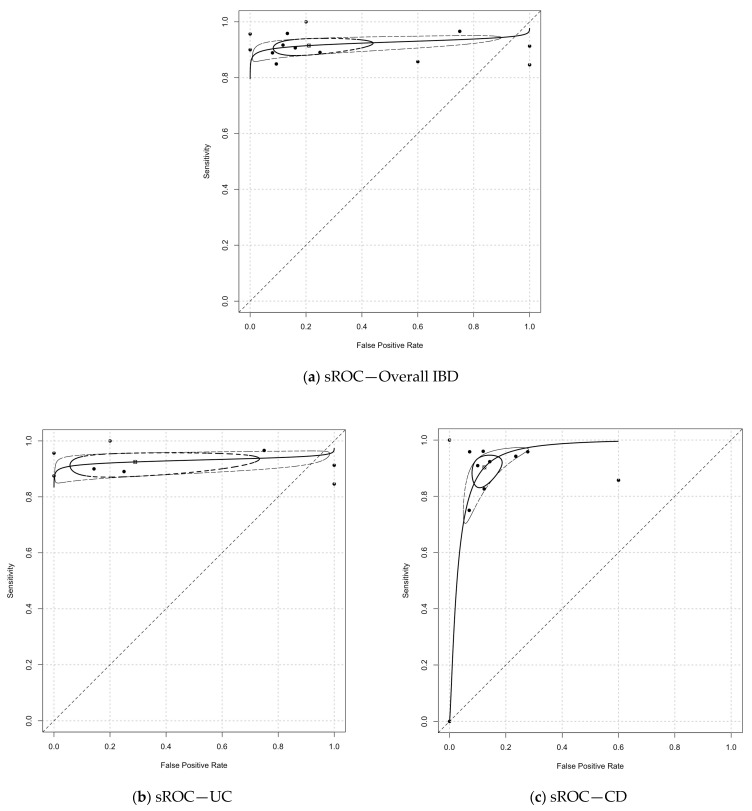
Summary receiver operating characteristic (sROC) curves of CCE for the diagnosis of (**a**) IBD overall, (**b**) ulcerative colitis, and (**c**) Crohn’s disease utilising the generalised linear mixed model (GLMM) from the “glmer” function in the R package “lme4”.

Table 1 presents the overall and subgroup analyses of CCE’s diagnostic test accuracy (DTA) in detecting IBD. Table 2 details the heterogeneity variance (τ^2^) and relative sensitivity and specificity as part of the heterogeneity assessment in conjunction with the subgroup analysis. Figure 2 illustrates the bivariate meta-analysis’s forest plot, highlighting CCE’s sensitivity and specificity in IBD. Figure 3 shows CCE’s summary receiver operating characteristic (sROC) curves in diagnosing IBD, UC, and CD.

The overall sensitivity and specificity of CCE in detecting IBD (both ulcerative colitis (UC) and Crohn’s disease (CD)) were 90% (95% CI, 85–93%) and 76% (95% CI, 56–90%), respectively. The sensitivity demonstrated low heterogeneity, with a τ^2^ of 0.13, while the specificity showed high heterogeneity, with a τ^2^ of 1.21. This suggests notable variability in specificity across the studies. An overall area under the curve (AUC) of 0.92 (95% CI, 0.94–0.97) indicates high diagnostic accuracy of CCE for IBD (see Figure 3).

In the subgroup analysis of UC (seven out of nine included studies), CCE’s pooled sensitivity was 92% (95% CI, 88–95%), with low heterogeneity (τ^2^ = 0.041). The pooled specificity, however, was 71% (95% CI, 35–92%), with substantial heterogeneity (τ^2^ = 3.56) and a wide confidence interval. The correlation coefficient between CCE and colonoscopy ranged from 0.75 to 0.86 across the six UC studies. Two studies contributed significantly to the heterogeneity in UC specificity, likely due to their small sample sizes (*n* < 30).

For CD (five out of nine included studies), the pooled per-patient sensitivity of CCE was 90% (95% CI, 85–93%), with a τ^2^ of 0.24, and the pooled specificity was 88% (95% CI, 83–91%), showing low heterogeneity (τ^2^ = 0.13). The AUC for CD detection was 0.87 (95% CI, 0.76–0.98), indicating a moderate diagnostic accuracy of CCE for CD compared with colonoscopy. The correlation coefficient between CCE and colonoscopy in CD studies ranged from 0.49 to 0.82. Further subgroup analysis comparing CD colitis with CD terminal ileitis revealed a sensitivity of 85% (95% CI, 77–90%) and a specificity of 90% (95% CI, 86–94%) for CD colitis compared with 95% (95% CI, 90–97%) and 84% (95% CI, 76–89%) for CD terminal ileitis. Both subgroups exhibited low heterogeneity (τ^2^), suggesting consistency in the results. The relative sensitivity of CCE in detecting CD colitis versus CD terminal ileitis was 0.89 (95% CI, 0.82–0.97), which was statistically significant, indicating its greater sensitivity in detecting terminal ileitis. The relative specificity, however, was not significant (1.08; 95% CI, 0.89–1.18), and the AUC for terminal ileitis (0.95) was higher than for colitis (0.86). The small number of patients and studies involving CD colitis and terminal ileitis limited the accuracy of the AUC 95% CI in the generalised linear mixed model (GLMM), as shown in Table 1.

Comparing UC and CD using CCE, the relative sensitivity was 1.02 (95% CI, 0.96–1.09), and the relative specificity was 0.81 (95% CI, 0.52–1.26), suggesting that CCE has better specificity for CD, although this was not statistically significant. Subgroup analysis of CCE1 versus CCE2 among the UC studies (as no CD study used CCE1) indicated a sensitivity of 89% (95% CI, 82–94%) and a specificity of 46% (95% CI, 44–94%) for CCE1 compared with 95% (95% CI, 86–98%) and 93% (95% CI, 17–99%) for CCE2. Although the relative specificity of CCE1 compared with CCE2 was 0.49 (95% CI, 0.1–2.4), technological advancements likely contributed to the improved accuracy observed with CCE2 despite the difference not reaching statistical significance.

When comparing CCE2 to Crohn’s disease capsules, the subgroup difference in relative sensitivity (0.95; 95% CI, 0.86–1.04) and relative specificity (1.07; 95% CI, 0.96–1.18) showed no statistical significance, suggesting comparable accuracy in IBD detection in both the terminal ileum and the colon. Additional subgroup analyses on completion rates, the use of prokinetics, and bowel preparation regimens revealed no statistically significant differences. This is mainly due to the considerable variability in bowel preparation regimens and booster protocols. Consequently, this emphasises an area deserving of more future research.

## 4. Discussion

Despite the increased acceptance of CCE, the lack of data has made it difficult to position it as a routine lower GI investigation modality globally. Therefore, this current study is an updated systematic review of CCE and the first meta-analysis on CCE in inflammatory bowel disease and other non-polypoidal colonic inflammatory conditions [9].

Based on our meta-analysis, CCE demonstrates a diagnostic accuracy comparable to colonoscopy, with a pooled sensitivity of 90% in detecting IBD overall, 92% in UC disease, and 90% in CD disease activity. The overall AUC was 0.92 in IBD, 0.93 in UC, and 0.87 in CD. These aligned with the findings by Tamilarasan et al. on panenteric capsule endoscopy. It is worth noting that the variance of specificity for IBD overall was 1.21, which is likely attributed to UC’s high variance of specificity of 3.56. This suggests that there was significant heterogeneity in the UC pooled specificity. From analysing the forest plot, the two small studies (Ye et al. [28], *n* = 25; Meister et al. [34], *n* = 13) are the most likely cause of this heterogeneity. After excluding the two small studies in the post hoc subgroup analysis, the heterogeneity variance of UC improved to 2.12 from 3.56, the pooled UC specificity improved to 82% (95% CI, 51–96%) from 71%, and the AUC improved from 0.93 to 0.94. This improvement underscores the importance of adequate sample sizes and power calculations during the planning phase of studies. 

Retrospective power calculations were conducted to evaluate the diagnostic accuracy of CCE, mainly focusing on its pooled sensitivity and specificity relative to colonoscopy. Assuming a true sensitivity and specificity of 0.9 for both UC and CD and using an alpha level of 0.05 with a desired power of 0.9, the calculations (performed using the “pwr” function within the “metafor” package) indicated that each study should ideally have had a minimum sample size of 27, with at least six studies and a total sample size of 162 required to achieve the desired statistical power. Nevertheless, given the limited number of CCE studies on IBD, studies with a minimum sample size of 10 were included in the primary analysis to ensure sufficient studies for an adequate meta-analysis.

In the context of Crohn’s disease, the diagnostic yield of CCE also appeared to be comparable to ileocolonoscopy on the per-patient level, with a low value of heterogeneity. In the subgroup analysis, a lower relative sensitivity of 0.89 was observed when comparing CD colitis to terminal ileitis. This statistically significant finding suggests reduced sensitivity in detecting CD colitis, likely due to the inherent challenges of precise lesion localisation in CCE, particularly around the colonic flexures [48]. While the detection of inflammation posed no significant challenges, difficulty arises in the uncertainty when correlating these findings to those obtained from IC. 

When comparing different capsule iterations, CCE2 showed a significant improvement in specificity compared with CCE1 and a marginal increase in Crohn’s capsule compared with CCE2; there was no statistical difference in the relative sensitivity and specificity when comparing the different models. The improvement could be due to software technology, as described by Tamilarasan et al. and Nia et al. [9,49]. However, the number of studies in these subgroups was very small, indicating the need for more diagnostic accuracy within this area of research. 

Furthermore, while IC stands as the gold standard, its accuracy in detecting small-bowel CD is surpassed by 22% in the diagnostic yield compared with small-bowel CE, as reported in the systematic review conducted by Dionisio et al. [50]. Employing an imperfect gold standard for comparing diagnostic accuracy with CCE, especially in terminal ileitis, raises some concerns. The occurrences of false positives in CCE, especially in the terminal ileum, might indicate cases overlooked by colonoscopy. This introduces a potential source of discrepancy that could diminish CCE’s perceived accuracy despite its potential for a superior diagnostic yield. Therefore, caution must be considered when interpreting the results of diagnostic assessments, particularly in terminal ileal disease. The suggestion by Bruining et al. for addressing this potential challenge was to adopt a panel consensus with the discrepancies. This involves reviewing and discussing the panellists’ endoscopic videos, laboratory results, and clinical notes to confirm the findings and secure the diagnosis. Another included study by Leighton et al. took a distinctive approach by presenting the diagnostic yield of Crohn’s disease without using IC as the gold standard. However, it regarded capsule finding as an endpoint diagnosis, acknowledging the argument above. This led to a 16% improvement in the diagnostic yield of terminal ileitis in CCE compared with IC [46]. However, a drawback of this approach lies in the poor specificity of ileal ulcers, especially in the absence of histological confirmation. This might account for the observed inferior specificity of CCE in Crohn’s terminal ileitis compared with its colonic counterpart.

Furthermore, another critical challenge arises from the uncertainty around the definition of terminal ileum. A recent descriptive study revealed a range of 1 cm to 17 cm of terminal ileum specimens from surgical resections [51]. Another small study also showed that the average length of the examined terminal ileum by colonoscopy was 12.93 ± 6 cm, demonstrating a significant variation in the definition [52]. In clinical practice, the precise measurement of the last 10 cm of the terminal ileum poses a considerable challenge to CCE [46]. This might contribute to the statistically significant increase in CCE’s sensitivity in detecting terminal ileal Crohn’s disease.

Nevertheless, the AUC for the use of CCE in Crohn’s terminal ileitis (0.95) is better than that in Crohn’s colitis (0.86), accepting the potential limitation of the small number of patients and studies. We can only postulate that the reason for the better diagnostic accuracy in the TI might be the more stable capsule’s movement in a smaller lumen and generally better bowel preparation in the terminal ileum compared with its unpredictable rocking motions in the colon and the higher risk of poor colonic bowel preparation [26,46].

Interstudy heterogeneity remains a significant challenge for analysis and data interpretation. This is attributed to different disease activity assessment scores and analysis methods (e.g., per-patient, per-segment, per-lesion, and per-characteristics). Other challenges include the use of diverse correlation coefficients, the uncertainty in securing the diagnosis of Crohn’s disease without an adequate follow-up period, and a decent gold standard reference for comparison. The exclusion of patients with acute severe ulcerative colitis due to the requirement of urgent inpatient investigation and treatment, as well as the deliberate avoidance of individuals with Crohn’s disease with severe stricturing (to mitigate the risk of capsule retention), may introduce a potential selection bias that could inadvertently favour CCE with less-adverse events. In addition, the absence of a pre-registered protocol for this systematic review and meta-analysis might introduce some potential limitations in selective reporting biases and transparency. Ultimately, the inability of CCE to acquire biopsies for further histological assessment remains the primary constraint in IBD assessment, especially in the context of dysplasia detection in UC and the identification of malignancy in this higher-risk patient cohort. However, the increasingly encouraging results of AI in CCE may offer a future solution for detecting dysplasia and subtle malignancies, potentially reducing or eliminating the need for biopsies [27].

During the literature search, the limited number of comparative studies on non-polypoidal inflammation (e.g., infective colitis, checkpoint inhibitor colitis, or diverticulitis) highlighted a significant gap in the research. 

## 5. Conclusions

This systematic review and meta-analysis demonstrated that CCE is comparable to colonoscopy in diagnostic yield in diagnosing UC, Crohn’s terminal ileitis, and colitis in the context of adequate bowel preparation and the procedure completion rate. It has a high sensitivity for both UC and Crohn’s disease, which suggests its possible use as a screening tool, for example, in patients with elevated faecal calprotectin or high risk of IBD. These findings can also guide further appropriate investigations. Regarding UC disease activity assessment, it is unclear whether CCE would have any additional value to faecal calprotectin and colonoscopy, but it could be utilised as an alternative. However, CCE might be useful in assessing the distribution of CD, especially in the terminal ileum where it eludes colonoscopic reach. Further studies are required to evaluate the diagnostic accuracy of other non-polypoidal colonic pathologies.

## Figures and Tables

**Table 1:** Overall and subgroup analysis for the diagnostic accuracy of CCE in IBD using a generalised linear mixed model (GLMM).

Overall and Subgroup Analysis	Pooled Sensitivity (95% CI)	Pooled Specificity (95% CI)	Pooled PLR (95% CI)	Pooled NLR (95% CI)	Pooled DOR (95% CI)	SROC-AUC (95% CI)
IBD overall (*n* = 13)	0.90 (0.85–0.93)	0.76 (0.56–0.90)	5.43 (5.39–5.46)	0.107 (0.106–0.108)	50.79 (50.27–51.30)	0.92 (0.94–0.97)
No prokinetics (*n* = 3)	0.87 (0.77–0.93)	0.71 (0.27–0.94)	3.04 (−1.07–7.13)	0.18 (0.058–0.31)	16.64 (−14.10–47.37)	NA (*n* < 5)
Metoclopramide (*n* = 4)	0.93 (0.89–0.96)	0.76 (0.58–0.88)	3.86 (1.50–6.22)	0.09 (0.051–0.13)	42.92 (10.88–74.95)	NA (*n* < 5)
Domperidone (*n* = 4)	0.91 (0.84–0.95)	0.93 (0.71–0.99)	13.33 (−7.89–34.56)	0.099 (0.044–0.15)	134.67 (−108.65–377.99)	NA (*n* < 5)
UC pooled (*n* = 7)	0.92 (0.88–0.95)	0.71 (0.35–0.92)	3.19 (−0.24–6.62)	0.11 (0.049–0.16)	30.16 (−14.11–74.43)	0.93 (0.89–0.97)
CCE1 capsule (*n* = 4)	0.89 (0.82–0.93)	0.46 (0.44–0.94)	1.64 (−0.54–3.83)	0.24 (−0.16–0.64)	6.89 (−13.61–27.38)	NA (*n* < 5)
CCE2 capsule (*n* = 4)	0.95 (0.86–0.98)	0.93 (0.17–0.99)	14.54 (−42.6–7.17)	0.052 (0.0078–0.096)	279.62 (−859.73–1419)	NA (*n* < 5)
CD pooled (*n* = 6)	0.90 (0.85–0.93)	0.88 (0.83–0.91)	7.30 (4.90–9.69)	0.11 (0.061–0.16)	65.75 (30.96–100.55)	0.87 (0.76–0.98)
CCE2 capsule (*n* = 4)	0.88 (0.79–0.93)	0.90 (0.84–0.93)	8.74 (5.16–12.31)	0.13 (0.056–0.21)	66.45 (18.34–114.56)	NA (*n* < 5)
Crohn’s capsule (*n* = 2)	0.93 (0.88–0.96)	0.85 (0.76–0.91)	6.07 (3.21–8.92)	0.08 (0.032–0.13)	75.5 (11.45–139.47)	NA (*n* < 5)
CD colitis (*n* = 6)	0.85 (0.77–0.90)	0.90 (0.86–0.94)	8.93 (5.19–12.68)	0.17(0.093–0.24)	54.85 (17.93–89.76)	0.86 (*n* = too small)
CD terminal ileitis (*n* = 5)	0.95 (0.90–0.97)	0.84 (0.76–0.89)	5.89 (3.49–8.29)	0.059 (0.018–0.10)	99.18 (12.95–185.40)	0.95 (*n* = too small)

**Table 2:** Relative sensitivity and specificity and subgroup heterogeneity in sensitivity and specificity using generalized linear mixed models (GLMMs).

Overall and Subgroup	Parameter	Logit Scale		Back-Transformed		Heterogeneity Variance τ^2^	Relative Sensitivity and Specificity	Estimate	95% CI
		Mean	Standard Error	Estimate	95% CI				
IBD overall (*n* = 13)	Sensitivity	2.33	0.0037	0.90	0.85–0.93	0.13	NA (only applicable for subroup analysis)	NA	NA
	Specificity	1.60	0.0037	0.76	0.56–0.90	1.21			
No prokinetics (*n* = 3)	Sensitivity	1.90	0.35	0.87	0.77–0.93	0.33	NA (non-dichotomous comparators)	NA	NA
	Specificity	0.91	0.98	0.71	0.27–0.94	0.16			
Metoclopramide (*n* = 4)	Sensitivity	2.613	0.27	0.93	0.89–0.96	0.37	NA (non-dichotomous comparators)	NA	NA
	Specificity	1.146	0.42	0.76	0.58–0.88	0.97			
Domperidone (*n* = 4)	Sensitivity	2.29	0.30	0.91	0.84–0.95	0.00	NA (non-dichotomous comparators)	NA	NA
	Specificity	2.62	0.87	0.93	0.71–0.99	1.39			
UC pooled (*n* = 7)	Sensitivity	2.51	0.25	0.92	0.88–0.95	0.04	UC/CD Relative Sens	1.02	0.96–1.09
	Specificity	0.90	0.78	0.71	0.35–0.92	3.56			
CD pooled (*n* = 6)	Sensitivity	2.23	0.26	0.90	0.85–0.93	0.24	UC/CD Relative Spec	0.81	0.52–1.26
	Specificity	1.96	0.20	0.88	0.83–0.91	0.13			
CCE1 capsule (*n* = 4)	Sensitivity	2.10	0.30	0.89	0.82–0.94	<0.01	CCE1/CCE2 Relative Sens	0.94	0.87–1.01
	Specificity	–0.17	1.48	0.46	0.44–0.94	3.28			
CCE2 capsule (*n* = 4)	Sensitivity	2.97	0.47	0.95	0.86–0.98	0.16	CCE1/CCE2 Relative Spec	0.49	0.10–2.4
	Specificity	2.66	2.15	0.93	0.17–0.99	8.26			
Crohn’s capsule (*n* = 2)	Sensitivity	2.62	0.33	0.93	0.88–0.96	<0.01	CCE2/Crohn’s Cap Relative Sens	0.95	0.86–1.04
	Specificity	1.71	0.28	0.85	0.76–0.91	0.11			
CCE2 capsule (*n* = 4)	Sensitivity	2.01	0.33	0.88	0.79–0.93	0.21	CCE2/Crohn’s Cap Relative Spec	1.07	0.96–1.18
	Specificity	2.19	0.24	0.90	0.84–0.93	0.05			
CD colitis (*n* = 6)	Sensitivity	1.73	0.26	0.85	0.77–0.90	0.05	Colitis/TI Relative Sens	0.89	0.82–0.97 *
	Specificity	2.25	0.24	0.90	0.86–0.94	0.02			
CD terminal ileitis (*n* = 5)	Sensitivity	2.95	0.37	0.95	0.90–0.97	<0.01	Colitis/TI Relative Spec	1.08	0.98–1.18
	Specificity	1.65	0.25	0.84	0.76–0.89	0.03			

* Indicates that the subgroup is statistically significant.

**Figure A3:**
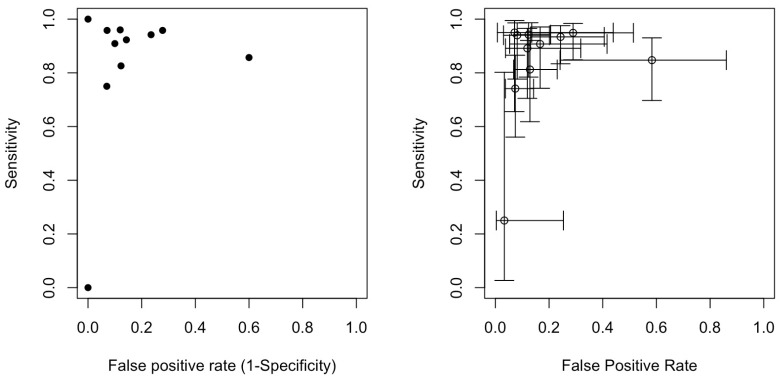
Scatterplots showing variations in the cut-off points as well as the accuracy of CCE in overall IBD diagnosis.

## Data Availability

The raw data supporting the conclusions of this article will be made available by the authors on request.

## Appendix A

This includes the search terms for the literature search, a summary of the key findings of the ulcerative colitis studies, an overview of the key findings of the Crohn’s disease studies, a summary of the CCE-associated regimens and information, a summary of the key findings of studies reporting other colonic pathology, the risk-of-bias assessments, the search strings used, and other forest plots of the subgroup analysis.

**Table A1:** The search terms for the literature search using the PICO framework. These terms were used in combination with Boolean operators and truncations to narrow the search, as appropriate, in addition to the MeSH terms search. For a comprehensive systematic review, search limits were not employed due to the concern that this could result in missing papers.

Domain	Search Terms
Population	Patients including both paediatric and adult participants; patients referred after positive FIT/faecal calprotectin or imaging testing; patients referred with lower gastrointestinal symptoms or assessment of their IBD.
Intervention	Colon capsule endoscopy (CCE)—Pillcam Colon Capsule 1 and 2
Comparison	Optical endoscopy: Ileocolonoscopy (IC) and transanal enteroscopy
Outcome	Inflammatory bowel disease
	Ulcerative colitis
	Crohn’s colitis
	Diverticulitis
	Infective colitis
	Microscopic colitis
	Autoimmune/checkpoint inhibitor colitis
	Lower GI haemorrhage/bleeding
	Haemorrhoids
	Telangiectasia
	Radiation colitis

**Table A2:** Summary of the key findings of the ulcerative colitis studies.

Ulcerative Colitis Publications								
Author and Location	Year	Study	UC and Comparator	Sample Size Included/Enrolled (Paediatric/Adult)	Accuracy	UC/CD Score	Correlation Coefficients for Disease Activity Detection	Disease Extent Agreement:
Ye C. A. et al., China [28]	2013	Prospective single-centre	UC Ileocolonoscopy	25/25 (adult)	100% sensitivity	Mayo score	Cohen κ = 0.751	-
Juan-Acosta et al., Spain [29]	2014	Prospective single-centre	UC Ileocolonoscopy	42/42 (adult)	Sensitivity 77.8% Specificity 95.8% PPV 93.3% NPV 85.19%	Mayo score	Cohen κ = 0.79 (CI: 0.62–0.96)	κ = 0.71 (CI: 0.52–0.90)
Shi et al., Hong Kong [30]	2017	Prospective single-centre	UC Ileocolonoscopy	108/150 (adult)	Sensitivity 94% Specificity 71% PPV 58% NPV 96%	Mayo and UCEIS scores	Intraclass correlation coefficient ICC > 0.8 Bleeding > 0.9 Ulcers > 0.8 Vascular pattern 0.77–0.95	-
Adler et al., Isreal [31]	2019	Prospective multicentre	UC Ileocolonoscopy	23/30 (adult)	Data available to calculate accuracy	Mayo score	Kappa coefficient κ = 0.86 Percent agreement = 95.7%	κ = 0.42
Oliva et al., Italy [32]	2014	Prospective single-centre	UC Ileocolonoscopy	29/30 (paediatric)	Sensitivity 96% (CI 79–99) Specificity 100% (CI 61–100) PPV 100% (CI 85–100) NPV 85% (CI 49–97) Accuracy 97%	Matts score	Cohen κ > 0.86	Paris classification E1 sensitivity 67% and specificity 100% E2 sensitivity 100% and specificity 95% E3 sensitivity 86% and specificity 100% E4 sensitivity 100% and specificity 100%
Sung et al., Hong Kong [33]	2012	Prospective multicentre	UC Ileocolonoscopy	96/100 (adult)	Sensitivity 89% Specificity 75% PPV 93% NPV 65%	-	-	-
Meister et al., Italy [34]	2013	Prospective single-centre	UC Ileocolonoscopy	13/13 (adult)	Data available to calculate accuracy	Rachmilewitz score	Kruskal–Wallis test comparing OC and CCE	-
Hosoe et al., Japan [35]	2013	Prospective single-centre	UC Ileocolonoscopy	29/30 (adult)	-	Matts endoscopic scores	Spearman’s rank coefficient ρ = 0.797	Caecum ρ = 0.862 Ascending ρ = 0.906 Transverse ρ = 0.778 Left side ρ = 0.765 Distal left side ρ = 0.673
Hosoe et al., Japan [36]	2018	Prospective single-centre	UC Ileocolonoscopy	22/38 (adult)	-	CSUC UCEIS	Spearman rank correlation 0.55 (CI 0.38–0.72)	-

**Table A3:** Summary of the key findings of the Crohn’s disease studies.

Crohn’s Disease Publications								
Author and Location	Year	Study	CD and Comparator	Sample Size Included/Enrolled (Paediatric/Adult)	Accuracy	UC/CD Score	Severity	Segmental Disease/Characteristics Correlation
Oliva et al., Italy [37]	2016	Prospective single-centre	CD Ileocolonoscopy	40/40 (paediatric)	Colon: Sensitivity 89%, specificity 100%, PPV 100%, and NPV 91% TI: Sensitivity 94%, specificity 100%, PPV 100%, and NPV 96%	SES-CD Lewis score	Interobserver agreement in disease activity within CCE only Cohen κ = 0.91	-
Brodersen et al., Denmark [38]	2022	Prospective multicentre	CD Ileocolonoscopy	130/153 (43 CCE2 and 90 Crohn’s capsule) (adult)	Colon: Sensitivity 75%, specificity 93%, PPV 71%, and NPV 93% TI: Sensitivity 96.6%, specificity 71.8%, PPV 98.7%, and NPV 96%	SES-CD Lewis score	Spearman r = 0.82 between CCE and IC	-
Hausmann et al., Germany [39]	2017	Prospective multicentre	CD Ileocolonoscopy	12/22 (adult)	Ileocolon post-surgical recurrence: Sensitivity 83% Specificity 100% PPV 100% NPV 90%	Rutgeerts score	-	-
Leighton et al., USA [46]	2017	Prospective multicentre	CD Ileocolonoscopy	66/114 (adult)	Diagnostic yield per subject 83.3%. Diagnostic yield per bowel segment 40.6% Detection rate: colon—34.4%, TI—54%	-	Kappa coefficient for agreement Per-subject yield κ = 0.384 Per-segment yield κ = 0.578	-
Bruining et al., USA [40]	2020	Prospective multicentre	CD Ileocolonoscopy	99/158 (adult)	Colon: Sensitivity 83%, specificity 88%, PPV 70%, and NPB 93% TI: Sensitivity 94%, specificity 81%, PPV 86%, and NPV 91%	SES-CD Lewis score	spearman’s coefficient and kappa coefficient for agreement TI κ = 0.579 (*p* < 0.001) COLON κ = 0.440 (*p* < 0.001)	-
Yamada et al., Japan [41]	2021	Prospective single-centre	CD Transanal double-balloon enteroscopy	20/22 (adult)	Per lesion: Colon: Sensitivity 85.7%, specificity 78.3%, PPV 32%, and NPV 97.8% TI: Sensitivity 90.2%, specificity 76.6%.	SES-CD CECDAI	-	-
Papalia et al., Australia [42]	2021	Prospective single-centre	CD Ileocolonoscopy	47/47 (adult)	-	SES-CD	overall agreement between CCE and IC κ = 0.49	Disease activity between CCE and IC: TI r = 0.77 (*p* = 0.001) Ascending r = 0.38 (*p* = 0.01) Transverse r = 0.43 (*p* = 0.01) Descending r = 0.46 (*p* = 0.001) Rectum r = 0.16 (*p* = 0.38)
Brodersen et al., Denmark (within the diagnostic study 2022) [43]	2023	Prospective multicentre	CD Ileocolonoscopy	99/99 (adult)	-	SES-CD	SES-CD score: Overall agreement ICC = 0.83 Spearman correlation coefficient, r = 0.78, *p* < 0.001)	Cohen κ for - ulcer size (κ = 0.46, *p* < 0.001); - ulcerated surface κ = 0.34, *p* < 0.001); - affected surface κ = 43, *p* < 0.001)
Hall et al., Ireland [47]	2015	Prospective single-centre	CD Ileocolonoscopy	10/10 (adult)	-	SES-CD	Interobserver agreement overall detection r = 0.6667, *p* < 0.035)	-
D’Haens et al. [44]	2015	Prospective multicentre	CD Ileocolonoscopy	40/40	Colon: Sensitivity 86%, specificity 40% TI: Data available to calculate the accuracy	SES-CD CDEIS CDAI GELS	Interobserver ICC: Overall = 0.65 Ileum = 0.73 Colon = 0.53 (average)	Interobserver ICC: Caecum = 0.6 Right colon = 0.61 Left colon = 0.43 Rectum = 0.49

**Table A4:** The summary of the CCE-associated regimens and additional information from all the included studies.

Additional Information from All Studies								
Author	Capsule Type	Bowel Preperation	Volume	Prokinetic	Booster	Volume	Bowel Cleansing Assessment	Completion Rate
Ismail M. et al., 2021 [53]	Pillcam Colon 2	PEG	2 L	-	Phospho-soda and gastrograffin	-	38% Good 55% Adequate 8% Inadequate	76% (50/66)
Ye C. A. et al., 2013 [28]	Pillcam Colon 1	PEG	2 L	-	Phospho-soda	-	80% Adequate	100%
Akyuz U et al., 2016 [54]	Pillcam Colon 2	PEG	4 L	Metoclopramide 10 mg TDS	Phospho-soda and bisacodyl suppository	45 mL and 5 mg	3 different bowel prep regimens	100%
Hosoe et al., 2014 [35]	Pillcam Colon 2	PEG	2 L	Metoclopramide once only	PEG and mosapride citrate	250 mL and 20 mg	42.5% Good <65% Fair	69%
Juan-Acosta et al., 2014 [29]	Pillcam Colon 1 and 2	Sennosides PEG	48 mg 4 L	Domperidone once only	Sodium phosphate	60 mL	32.5% Excellent 47.5% Good 15% Fair 5% Poor	85.7%
Meister et al., 2013 [34]	Pillcam Colon 1	PEG	1.75 L	Domperidone once only	PEG and bisacodyl suppository	750 mL and 10 mg	90% Adequate	77%
Shi et al., 2017 [30]	Pillcam Colon 2	PEG	4 L	Metoclopramide once only	Phosphate soda and bisacodyl suppository	45 mL and 10 mg	66% Adequate	68%
Sung et al., 2012 [33]	Pillcam Colon	PEG	4 L	Metoclopramide once only	Sodium phosphate and bisacodyl suppository	45 mL and 10 mg	7% Excellent 57% Good 31% Fair 4% Poor	83%
Hosoe et al., 2018 [36]	Pillcam Colon 2	PEG	0.7 L	Metoclopramide once only	Mosapride citrate and magnesium citrate	20 mg and 2.1 L	92% Adequate	69%
Oliva et al., 2014 [32]	Pillcam Colon 2	PEG	50 mL/kg (2 L max)	Domperidone once only	Sodium phosphate and bisacodyl suppository	45 mL and 10 mg	17% Excellent 45% Good 24% Fair 14% Poor	86%
Papalia et al., 2021 [42]	Pillcam Colon 2	PEG	2 L	Metoclopramide once only	PEG and bisacodyl suppository	500 mL and 10 mg	17% Excellent 30% Good 26% Fair 8% Poor	68%
Oliva et al., 2016 [37]	Pillcam Colon 2	PEG	Up to 2 L	Domperidone once only	Sodium phosphate and bisacodyl suppository	45 mL and 10 mg	21% Excellent 42% Good 26% Fair 11% Poor	95%
Brodersen et al., 2022 [38]	Pillcam Colon 2 and Crohn’s	PEG	4 L	Nil	Sodium phosphate and bisacodyl suppository	-	75% Excellent 1.5% Poor	82%
Hausmann et al., 2017 [39]	Pillcam Colon 2	PEG	4 L	Domperidone once only	Nil	-	91.6% Adequate	80%
Yamada et al., 2021 [41]	Pillcam Colon 2	Senna Magnesium Citrate PEG	4 tablets, 50 g 1 L	Mosapride citrate	PEG Castor oil Sodium picosulphate Magnesium citrate	1 L 60 mL 48 mg 50 g	80% Adequate	75%
Brodersen et al., 2023 [43]	Pillcam Colon 2 (33/99) and Crohn’s (66/99)	Bisacodyl PEG	10 mg 4 L	Nil	Sodium phosphate and bisacodyl suppository	55 mL and 10 mg	48% Excellent or Good	See diagnostic study 2022
Hall et al., 2015 [47]	Pillcam Colon 2	Senna PEG	4 L	Nil	Sodium picosulphate	2 sachets	-	-
Bruining et al., 2020 [40]	Crohn’s capsule	PEG	4 L	Metoclopramide or erythromycin	Sodium sulphate, potassium sulphate, and magnesium sulphate. Bisacodyl suppository or erythromycin	1 L and 10 mg or 250 mg	64% Adequate in the colon 90% Adequate in the TI	85.6%
Leighton et al., 2017 [46]	Crohn’s capsule	PEG	4 L	Metoclopramide once only	Suprep and bisacodyl suppository	176 mL and 10 mg	Good/Excellent: TI 97.9%; Colon 48.7%	92%
Adler et al., [31]	Crohn’s capsule	PEG	3 L	Metoclopramide once only	Sodium sulphate, potassium sulphate, and magnesium sulphate	264 mL	-	-
Eliakim R et al., 2006 [55]	Pillcam Colon 1	PEG	3 L	-	Sodium phosphate and bisacodyl suppository	15 mL and 10 mg	Good or Excellent 84%	78%
Herrerias-Gutierrez et al. [56]	Pillcam Colon 1	PEG	3 L	Domperidone once only	Sodium phosphate and bisacodyl suppository	75 mL and 10 mg	Good or Excellent 65.6% Fair 19.4% Poor 15%	93%
D’Haens et al., 2015 [44]	Pillcam Colon 2	Sennocol PEG	4 L	-	Phospho-soda	60 mL	Excellent 40% Good 44% Fair 16% Poor 0%	85%

**Table A5:** Summary of the key findings of studies reporting other colonic pathologies.

Other Colonic Pathology Publications						
Author and Location	Year	Study	Comparator	Other Pathology	Sample Size Included/Enrolled (Paediatric/Adult)	Accuracy
Ismail M. et al., Ireland [53]	2021	Prospective single-centre	Ileocolonoscopy	Diverticulosis Haemorrhoids	66/77 (adult)	Diverticulosis (*n* = 11): Sensitivity: 100% Specificity: 94.5% PPV: 79% NPV: 100% Haemorrhoid (*n* = 3): 0/3 detected by CCE
Akyuz U et al., Turkey [54]	2016	Prospective single centre	Ileocolonoscopy	Diverticulosis Haemorrhoids Angiodysplasia	28/62 (adult)	Diverticulosis (*n* = 1) 1/1 detected by CCE Haemorrhoid (*n* = 1) 1/1 detected by CCE Telangeictasia (*n* = 1) 1/1 detected by CCE
Eliakim R et al., Israel [55]	2006	Prospective multicentre	Ileocolonoscopy	Diverticulosis	30/91 (adult)	Diverticulosis (*n* = 30): Sensitivity: 78% Specificity: 76% PPV: 47% NPV: 93%
Herrerias-Gutierrez J et al., Spain [56]	2011	Prospective multicentre	Ileocolonoscopy	Diverticulosis	134/144	Diverticulosis (*n* = 63): 2 cases were missed by colonoscopy Telangeictasia (*n* = 15): 2 cases were missed by colonoscopy

**Table A6:** The exclusion of five diagnostic studies with inadequate data for data synthesis.

Author	Year	Study	Reason for Exclusion
Okabayashi et al. [57]	2018	Prospective study—CCE compared with previous CS findings in UC	The duration between CCE and CS was unclear and there were inadequate data for analysis.
Takano et al. [58]	2018	Prospective study—CCE compared with series colonoscopy findings in UC	Unable to derive the TPs, TNs, FPs, and FNs from the available inadequate data in the paper.
Adler et al. [59]	2011	Prospective study—CCE evaluation compared with conventional CS in UC	Unable to derive the TPs, TNs, FPs, and FNs from the available inadequate data on disease severity and extent.
Carvalho et al. [60]	2015	Prospective study—Panenteric mucosal healing assessment	The follow-up colonoscopy was delayed, and the data was inadequate for data extraction for diagnostic yield.
Hasan-Keslat H. [61]	2015	Prospective study—CCE vs. colonoscopy in Crohn’s patients	This was presented as a percentage of agreement instead of in terms of correlation or diagnostic yield.

### Appendix A.1. The Search Strings Used in Each Database:

#### Appendix A.1.1. Medline Ovid

((capsule endoscop*/or capsule endoscopy/or ((capsule* or videocapsule*) adj3 (colonoscop* or endoscop*)).ti,ab,tw. or (pillcam colon* or (pill adj cam*)).ti,ab,tw. or (pillcam* or (pill adj cam*)).ti,ab,tw.) AND ((exp Inflammatory Bowel Diseases/or (inflammatory adj bowel adj disease*).ti,ab,tw. or exp Colitis/or colitis, Ulcerative/or exp Crohn Disease/or ((ulcerative or infectious or microscopic or autoimmune or checkpoint or inhibitor or radiation) adj colitis).ti,ab,tw. or crohn* disease.ti,ab,tw. or IBD.ti,ab,tw.) OR (exp Diverticular Diseases/or (diverticular adj2 disease*).mp. or diverticulosis.ti,ab,tw. or diverculitis.ti,ab,tw.) OR (exp Telangiectasis/or Telangiectas*.mp. or colonic angiodysplasia.ti,ab,tw. or (spider adj vein*).ti,ab,tw.) OR (exp Gastrointestinal Hemorrhage/or ((gastrointestinal or (lower adj GI)) adj (hemorrhage or haemorrhage)).ti,ab,tw.)) AND NOT ((exp polyp/or polyp*.mp. or colonic polyp.ti,ab,tw.) OR (exp colorectal cancer/or ((colorectal or (colon adj rectal)) adj cancer).ti,ab,tw.) NOT (duodeno* or jejeno* ileo*).ti.)

#### Appendix A.1.2. Embase Ovid

((capsule endoscop*/or capsule endoscopy/or ((capsule* or videocapsule*) adj3 (colonoscop* or endoscop*)).ti,ab,tw. or (pillcam colon* or (pill adj cam*)).ti,ab,tw. or (pillcam* or (pill adj cam*)).ti,ab,tw.) AND ((exp Inflammatory Bowel Diseases/or (inflammatory adj bowel adj disease*).ti,ab,tw. or exp Colitis/or colitis, Ulcerative/or exp Crohn Disease/or ((ulcerative or infectious or microscopic or autoimmune or checkpoint or inhibitor or radiation) adj colitis).ti,ab,tw. or crohn* disease.ti,ab,tw. or IBD.ti,ab,tw.) OR (exp Diverticular Diseases/or (diverticular adj2 disease*).mp. or diverticulosis.ti,ab,tw. or diverculitis.ti,ab,tw.) OR (exp Telangiectasis/or Telangiectas*.mp. or colonic angiodysplasia.ti,ab,tw. or (spider adj vein*).ti,ab,tw.) OR (exp Gastrointestinal Hemorrhage/or ((gastrointestinal or (lower adj GI)) adj (hemorrhage or haemorrhage)).ti,ab,tw.))AND NOT ((exp polyp/or polyp*.mp. or colonic polyp.ti,ab,tw.) OR (exp colorectal cancer/or ((colorectal or (colon adj rectal)) adj cancer).ti,ab,tw.) NOT (duodeno* or jejeno* ileo*).ti.)

#### Appendix A.1.3. Cochrane

((MeSH descriptor: [Capsule Endoscopes] explode all trees) OR (MeSH descriptor: [Capsule Endoscopy] explode all trees) OR (((capsule* or videocapsule*) NEXT (colonoscop* or endoscop*)):ti,ab,kw) OR (pillcam* or (pill NEXT cam*)):ti,ab,kw AND ((MeSH descriptor: [Colonoscopy] explode all trees) OR (MeSH descriptor: [Intestine, Large] explode all trees) OR ((sigmoidoscop* or rectoscop* anoscop* or colonoscop*):ti,ab,kw))) AND ((MeSH descriptor: [Inflammatory Bowel Diseases] explode all trees) OR ((inflammatory NEXT bowel NEXT disease*):ti,ab,kw) OR (MeSH descriptor: [Colitis] explode all trees) OR (MeSH descriptor: [Colitis, Ulcerative] explode all trees) OR (MeSH descriptor: [Crohn Disease] explode all trees) OR (((ulcerative or infectious or microscopic or autoimmune or checkpoint or inhibitor or radiation) NEXT colitis):ti,ab,kw) OR ((crohn* disease OR IBD):ti,ab,kw) OR (MeSH descriptor: [Diverticular Diseases] explode all trees) OR ((diverticular NEXT disease*):ti,ab,kw) OR ((diverticulosis OR diverticulitis):ti,ab,kw) OR (MeSH descriptor: [Telangiectasis] explode all trees) OR ((telangiectas* or “colonic angiodysplasia”):ti,ab,kw) OR ((spider NEXT vein*):ti,ab,kw) OR (MeSH descriptor: [Gastrointestinal Hemorrhage] explode all trees) OR ((((gastrointestinal or (lower NEXTGI)) NEXT (hemorrhage or haemorrhage))):ti,ab,kw))

#### Appendix A.1.4. Pubmed

(capsule endoscope[MeSH Terms] OR capsule endoscopy[MeSH Terms] OR ((capsule*[Title/Abstract] OR videocapsule*[Title/Abstract]) AND (colonoscop*[Title/Abstract] OR endoscop*[Title/Abstract]) OR (capsule*[Text Word] OR videocapsule*[Text Word]) AND (colonoscop*[Text Word] OR endoscop*[Text Word]))) AND colonoscopy[MeSH Terms] OR large intestine[MeSH Terms] OR ((sigmoidoscop*[Title/Abstract] OR rectoscop*[Title/Abstract] OR anoscop*[Title/Abstract] OR colonoscop*[Title/Abstract]) OR (sigmoidoscop*[Text Word] OR rectoscop*[Text Word] OR anoscop*[Text Word] OR colonoscop*[Text Word])) AND (inflammatory bowel disease[MeSH Terms] OR ((inflammatory[Title/Abstract] AND bowel[Title/Abstract] AND disease*[Title/Abstract])) OR ((inflammatory[Text Word] AND bowel[Text Word] AND disease[Text Word])) OR colitis[MeSH terms] OR colitis, ulcerative[MeSH Terms] OR crohn disease[MeSH Terms] OR ((ulcerative[Title/Abstract] OR infectious[Title/Abstract] OR microscopic[Title/Abstract] OR autoimmune[Title/Abstract] OR checkpoint[Title/Abstract] OR inhibitor[Title/Abstract] OR radiation[Title/Abstract]) AND colitis[Title/Abstract]) OR ((ulcerative[Text Word] OR infectious[Text Word] OR microscopic[Text Word] OR autoimmune[Text Word] OR checkpoint[Text Word] OR inhibitor[Text Word] OR radiation[Text Word]) AND colitis[Text Word]) OR IBD[Title/Abstract] OR IBD[Text Word] OR ((diverticulosis[MeSH Terms] OR “diverticular disease”) OR ((diverticulosis[Title/Abstract] OR diverticulitis[Title/Abstract]))) OR ((diverticulosis[Text Word] OR diverticulitis[Text Word])) OR ((telangiectasia[MeSH Terms] OR telangiectas*) OR (“colonic angiodysplasia”[Title/Abstract] OR spider vein*[Title/Abstract])) OR (“colonic angiodysplasia”[Text Word] OR spider vein*[Text Word]) OR ((gastrointestinal hemorrhage[MeSH Terms]) OR ((gastrointestinal[Title/Abstract] OR “lower GI”[Title/Abstract]) AND (hemorrhage[Title/Abstract] OR haemorrhage[Title/Abstract]))) OR ((gastrointestinal[Text Word] OR “lower GI”[Text Word]) AND (hemorrhage[Text Word] OR haemorrhage[Text Word]) NOT duodeno*[Title] OR ileo*[Title])
