# Correction: Lei et al. The Diagnostic Accuracy of Colon Capsule Endoscopy in Inflammatory Bowel Disease—A Systematic Review and Meta-Analysis. *Diagnostics* 2024, *14*, 2056

**DOI:** 10.3390/diagnostics15243222

**Published:** 2025-12-17

**Authors:** Ian Io Lei, Camilla Thorndal, Muhammad Shoaib Manzoor, Nicholas Parsons, Charlie Noble, Cristiana Huhulea, Anastasios Koulaouzidis, Ramesh P. Arasaradnam

**Affiliations:** 1 Institute of Precision Diagnostics & Translational Medicine, University Hospital of Coventry and Warwickshire, Clifford Bridge Rd, Coventry CV2 2DX, UK; cristiana.huhulea@uhcw.nhs.uk (C.H.); r.arasaradnam@warwick.ac.uk (R.P.A.); 2 Warwick Medical School, University of Warwick, Coventry CV4 7AL, UK; 3 Surgical Research Unit, Odense University Hospital, 5700 Svendborg, Denmark; camilla.thorndal.nielsen@rsyd.dk (C.T.); akoulaouzidis@hotmail.com (A.K.); 4 Department of Gastroenterology, Sandwell and West Birmingham Hospitals NHS Trust, Hallam St., West Bromwich B71 4HJ, UK; muhammad_shoaib_manzoor@yahoo.com; 5 Warwick Clinical Trials Unit, University of Warwick, Coventry CV4 7AL, UK; nick.parsons@warwick.ac.uk; 6 Noblesoft, Stamford, Lincs PE9 3DN, UK; c.noble@noblesoft.co.uk; 7 Department of Clinical Research, University of Southern Denmark, 5230 Odense, Denmark; 8 Department of Gastroenterology, Pomeranian Medical University, 70-204 Szczecin, Poland; 9 Department of Surgery, OUH Svendborg Sygehus, 5700 Svendborg, Denmark; 10 Leicester Cancer Centre, University of Leicester, Leicester LE1 7RH, UK

## Abstract Correction

There was an error in the abstract of the original publication [1]. The original publication states: “Overall, for inflammatory bowel disease, the pooled sensitivity, specificity, and AUC were 90% (95% CI, 85–93%), 76% (95% CI, 56–90%), and 0.92 (95% CI, 0.94–0.97), respectively”. A correction has been made in the abstract as follows:

“Overall, for inflammatory bowel disease, the pooled sensitivity, specificity, and AUC were 90% (95% CI, 85–93%), 76% (95% CI, 56–90%), and 0.92 (95% CI, 0.87–0.97), respectively”.

## Text Correction

There was an error in Section 3.5, paragraph 2 in the original publication [1]. The original publication states: “An overall area under the curve (AUC) of 0.92 (95% CI, 0.94–0.97) indicates high diagnostic accuracy of CCE for IBD (see Figure 3)”. A correction has been made as follows:

“An overall area under the curve (AUC) of 0.92 (95% CI, 0.87–0.97) indicates high diagnostic accuracy of CCE for IBD (see Figure 3)”.

## Error in Table 1

In the original publication [1], there was a mistake in Table 1: 0.92 (0.94–0.97). The corrected Table 1 appears below.

**Table 1:** Overall and subgroup analysis for the diagnostic accuracy of CCE in IBD using a generalised linear mixed model (GLMM).

Overall and Subgroup Analysis	Pooled Sensitivity (95% CI)	Pooled Specificity (95% CI)	Pooled PLR (95% CI)	Pooled NLR (95% CI)	Pooled DOR (95% CI)	SROC-AUC (95% CI)
IBD overall (*n* = 13)	0.90 (0.85–0.93)	0.76 (0.56–0.90)	5.43 (5.39–5.46)	0.107 (0.106–0.108)	50.79 (50.27–51.30)	0.92 (0.87–0.97)
No prokinetics (*n* = 3)	0.87 (0.77–0.93)	0.71 (0.27–0.94)	3.04 (−1.07–7.13)	0.18 (0.058–0.31)	16.64 (−14.10–47.37)	NA (*n* < 5)
Metoclopramide (*n* = 4)	0.93 (0.89–0.96)	0.76 (0.58–0.88)	3.86 (1.50–6.22)	0.09 (0.051–0.13)	42.92 (10.88–74.95)	NA (*n* < 5)
Domperidone (*n* = 4)	0.91 (0.84–0.95)	0.93 (0.71–0.99)	13.33 (−7.89–34.56)	0.099 (0.044–0.15)	134.67 (−108.65–377.99)	NA (*n* < 5)
UC pooled (*n* = 7)	0.92 (0.88–0.95)	0.71 (0.35–0.92)	3.19 (−0.24–6.62)	0.11 (0.049–0.16)	30.16 (−14.11–74.43)	0.93 (0.89–0.97)
CCE1 capsule (*n* = 4)	0.89 (0.82–0.93)	0.46 (0.44–0.94)	1.64 (−0.54–3.83)	0.24 (−0.16–0.64)	6.89 (−13.61–27.38)	NA (*n* < 5)
CCE2 capsule (*n* = 4)	0.95 (0.86–0.98)	0.93 (0.17–0.99)	14.54 (−42.6–7.17)	0.052 (0.0078–0.096)	279.62 (−859.73–1419)	NA (*n* < 5)
CD pooled (*n* = 6)	0.90 (0.85–0.93)	0.88 (0.83–0.91)	7.30 (4.90–9.69)	0.11 (0.061–0.16)	65.75 (30.96–100.55)	0.87 (0.76–0.98)
CCE2 capsule (*n* = 4)	0.88 (0.79–0.93)	0.90 (0.84–0.93)	8.74 (5.16–12.31)	0.13 (0.056–0.21)	66.45 (18.34–114.56)	NA (*n* < 5)
Crohn’s capsule (*n* = 2)	0.93 (0.88–0.96)	0.85 (0.76–0.91)	6.07 (3.21–8.92)	0.08 (0.032–0.13)	75.5 (11.45–139.47)	NA (*n* < 5)
CD colitis (*n* = 6)	0.85 (0.77–0.90)	0.90 (0.86–0.94)	8.93 (5.19–12.68)	0.17 (0.093–0.24)	54.85 (17.93–89.76)	0.86 (*n* = too small)
CD terminal ileitis (*n* = 5)	0.95 (0.90–0.97)	0.84 (0.76–0.89)	5.89 (3.49–8.29)	0.059 (0.018–0.10)	99.18 (12.95–185.40)	0.95 (*n* = too small)

The authors state that the scientific conclusions are unaffected. This correction was approved by the Academic Editor. The original publication has also been updated. 
